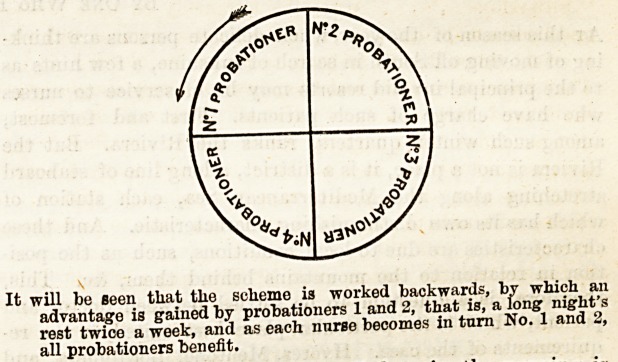# The Hospital Nursing Supplement

**Published:** 1896-11-14

**Authors:** 


### "The Hospital" Nursing Mirror.

Being the Extra Nursing Supplement of "The Hospital."

[Contributions for this Supplement should be addressed to the Editor, The Hospital, 28 & 29, Southampton Street, Strand, London, W.C., and should have the word "Nursing" plainly written in left-hand top corner of the envelope.]

### News from the Nursing World.

### THE NURSES AND QUEEN VICTORIA.

We are glad to hear that the nurses of the Royal National Pension Fund for Nurses have determined to make a special effort to commemorate the sixtieth year of Her Majesty's reign by increasing the Junius S. Morgan Benevolent Fund, the Benevolent Branch of the Royal National Pension Fund for Nurses, to a sum adequate to meet the claims of all nurses entitled to its benefits, who, through no fault of their own, may be in need of assistance. The feature we like best about this movement is that by which every member of the Pension Fund is spontaneously subscribing at least one shilling per annum to their Benevolent Branch. We hope, therefore, every nurse who has not yet obtained a collecting book should at once write to the Secretary, Mr. Louis Dick, 28, Finsbury Pavement, as this is a movement which every nurse can cordially join.

### DUNDEE SICK POOR NURSING SOCIETY.

A very interesting report of the good work done by the Dundee Sick Poor Nursing Society was read at the seventh annual meeting. It has been a year of exceptionally hard work, the directors reporting that the labours of the nurses were ever growing, and they felt could only stop when all the sick poor of the city were being attended to by trained nurses. The report of the inspector of the Queen's Jubilee Nurses' Institute, with which the society is affiliated, was eminently satisfactory, containing congratulations on the "great progress" made during the last few years, and on the "highly satisfactory position of the association and of the good work done by the nurses." Sir John Leng, M.P., who was in the chair, spoke with special appreciation of the work of the society, and very kindly of the nurses, urging those who could to help to "cheer, refresh, and encourage them," not only by words, but by deeds of kindness and sympathy, by sending them now and again flowers, or books, or inviting them to spend an hour or two away from the sometimes painful duties that daily devolved upon them. Dr. MacEwan, in seconding the adoption of the report, spoke of the importance of nursing and of its great modern advancement, and urged the strong claim of the society upon the sympathy of his hearers, hoping that no abatement, but rather an increase, of interest would be taken in its work in the future.

### NURSES AT GOVERNMENT HOUSE, MELBOURNE.

Lord and Lady Brassey gave an entertainment in the beginning of October at Government House, Melbourne, to what must have been a somewhat mixed multitude of the head and assistant teachers of the State schools, teachers in the Roman Catholic schools, some of the nurses of the various hospitals and convalescent homes, and the captains of the vessels in port. We quote from the *Argus* of October 5th. The plays acted were "Good for Nothing" and "The Duchess of Bayswater and Co.," in which the principal parts were taken by the Earl of Shaftesbury, Colonel Bingham, Mrs. Cornish, Miss Street, and Mr. J. L. Lilley. We are informed that the crush was very great, and many of the nurses present amongst the guests were prevented from getting any refreshment, a fact which must have been far from the intention of the host and hostess.

### ALCOHOL AND BREAST-FED INFANTS.

At the Paris Academy of Medicine on October 20th Dr. Nallin stated that infants at the breast are sometimes exposed to serious danger from their nurses indulging in alcohol to excess. It seems to be the habit in Paris to give a very liberal supply of wine or beer to the wet-nurses, by whom the infants of well-to-do families are so commonly brought up, and it is held that in some cases infantile convulsions and other nervous symptoms are caused by this alcohol, which is taken in excess, passing into the milk, and that they cease so soon as the nurse gives up her alcoholic beverages.

### IN THE CAUSE OF CHARITY.

The youngest daughter of Nathaniel Hawthorne, author of the well-known "Scarlet Letter" and "The House with the Seven Gables," has established herself in one of the poorest parts of New York City, intending to devote her life to visiting in their own homes those who are dying and hopelessly ill. Mrs. Lathrop's wish is specially to succour women dying of cancer, who are unable to find admission into any home for incurables, but many and various are the other claims upon her sympathy. The district nurses of New York will find a valuable friend in Mrs. Lathrop, one who can supplement their efforts for the relief of the sick poor in a most helpful manner.

### DIFFICULTIES AT SHEPTON MALLET.

Difficulties have arisen at the Shepton Mallet Infirmary between the nurse and the master and matron, and without commenting upon the cause of the dispute, which appears to be a very trivial one, it is significant to note that while the present master and matron have only been at the workhouse a few months, during which time (as was pointed out at a recent meeting of the guardians) six officers have left, nurse Harris has been there for three years, one of the guardians quoting in her favour a remark of a former patient that "he had never met a kinder nurse." Nurse Harris came before the board and resigned her appointment, and was told she could demand a Local Government Board inquiry, a step which she should certainly take in her own interest, as well as in that of all nurses who are suffering under the present unworkable system whereby untrained workhouse matrons are given authority over the nursing department.

### RUNCORN DISTRICT NURSE FUND.

The committee of the Runcorn District Nurse Fund reported at the seventh annual meeting recently held in the Council Chamber of the Town Hall, that "in the very competent hands of Nurse Buckingham the work had progressed in all respects satisfactorily." The fund possesses two good friends in Miss Pierpoint and Mrs. Soulby, whose kindness in inaugurating a fund for sending convalescents to homes or to country lodgings had been an invaluable aid to the nurse's work. The chairman, the Rev. Canon Maitland Wood, spoke at the meeting of the efforts which were being made to collect funds to erect a cottage hospital for the district, and reminded his audience that an income to maintain the institution would also be wanted. He mentioned this, he said, because it had been suggested that the district nurse should live at the hospital and take charge of the patients, and he thought it ought to be generally understood that it would be impossible for one woman, however zealous, to undertake this double work. It would be necessary to have another nurse if things were to be conducted satisfactorily—good advice, which the promoters of the cottage hospital scheme will be wise to lay to heart.

### SISTER JOSEPHINE'S FAREWELL TO DONCASTER.

Sister Josephine Gelston, who is just retiring from the post of Lady Superintendent of the Doncaster Infirmary, has held that appointment for nineteen years, and has been connected with the Midland Nursing Institute for twenty-four. The recent annual meeting was made the occasion of presenting Sister Josephine with an address on behalf of the committee and friends of the infirmary, expressing their high appreciation of the manner in which she had discharged the duties devolving upon her, and asking her acceptance of a purse of £90 as a token of "widespread regard." Miss Dean, Superintendent of the Mildmay Institute, who was present, afterwards presented Sister Josephine with a gold watch and chain from the directress, Mrs. C. Hogg, herself, and others, and the sisters and nurses of the Mildmay Home, as a mark of appreciation and love on her retirement "after twenty-four years of loyal connection with Mildmay," and with earnest affectionate wishes for her future happiness. Sister Josephine returned thanks in a few warm words of gratitude for the kind things said of her. She would carry back to Ireland very pleasant thoughts of the years spent in Doncaster. Sister Josephine's successor at the infirmary is another Mildmay sister, Sister Florence Longrigg.

### DISTRICT NURSING IN IRELAND.

Miss Dunn, Superintendent of the Irish branch of the Queen Victoria Jubilee Institute, addressed a meeting recently held at Swords, county Dublin, to consider the advisability of obtaining a nurse from the St. Lawrence's Home for the District. Miss Dunn explained at length the objects of district work; and the Rev. D. P. Mulcahy, and Dr. Fullam, medical officer for the district, urged the advantages of the scheme, which would be of the greatest service to the poor. A resolution was carried to provide a Queen's Nurse for Swords and Donabate, and a subscription list opened at once.

### DISTRICT NURSING FOR THE VALE OF LEVEN.

The late Mr. H. M. R. Ewing, whose name has been for years well known in Dumbartonshire, left a bequest of £2,000 to be invested for the benefit of Bonhill parish, and a number of ladies in the neighbourhood have been urging the formation of a district nursing association through its means. A meeting was held last month at which it was agreed to carry out this plan, to call the association the "H. M. R. Ewing District Nursing Association," and to make application to the Scottish Council for the services of a Queen's Nurse, and for affiliation. Mr. Ewing's trustees have agreed to hand over the interest of the money for three years to begin with. The matter has been taken up enthusiastically, and Mrs. Adair Campbell, who has been appointed president, was able to announce at the meeting annual subscriptions to the amount of £47, and donations of £41. Mrs. Campbell read at the meeting a paper by Miss Guthrie Wright on the subject of nursing associations.

### CHRISTMAS GIFTS FOR THE HOSPITALS.

Although we are not proposing to offer prizes for a needlework competition this winter, we shall be very glad if any contributions of warm and useful garments from our readers and their friends for the usual distribution amongst the sick poor in the London hospitals at Christmas time. Warm petticoats and nightgowns, flannel bed-jackets and nightingales, stockings, shirts, and so forth will be most welcome, and we feel sure that the kind friends who have sent us such nice things in this way on former occasions will not forget this year also to swell the number of serviceable gifts in The Hospital Christmas bundles, which give so much pleasure to those who are ill and destitute. Private nurses have many opportunities not only of giving a little of their own time for this purpose, but of interesting their patients or their patients' friends in this attempt to supply the poorest hospital patients with those warm necessaries without which rich invalids may well imagine the miseries of illness are increased tenfold. There are some people who can spare money better than time, and others to whom the reverse applies. Now, if those who can afford to buy material will do so, and find friends with more leisure than means to make them up into clothing for our Christmas bundles, a splendid result will be obtained, and we shall be more than glad to expedite matters by putting such helpers into communication if they will write to us on the subject. To this suggestion we invite our readers' earnest attention, and trust that bulky parcels will soon begin flowing into The Hospital offices, 28 & 29, Southampton Street, Strand, London, W.C., containing any and every sort of comfortable garment.

### SHORT ITEMS.

Nurse Martin, who has worked for nine years on the staff of the Edinburgh Royal Infirmary, and has just been appointed Lady Superintendent of the West Highland Hospital at Oban, was presented on leaving with a silver tea service from her friends and fellow-workers at the infirmary.—H.R.H. Princess Christian distributed medallions and certificates to the members of the Norwood Centre of the St. John Ambulance Association, of which she is President, at the Crystal Palace, on Friday, October 30th.—Very successful concerts were lately held in the Philharmonic Hall, at Southampton, in aid of the local branch of the Queen's Jubilee Nurses' Institute. Both afternoon and evening entertainments were largely attended.—The Grosvenor Hospital for Women and Children, Vincent Square, Westminster, has received twenty guineas from the Worshipful Company of Merchant Taylors.—A new wing to the Sheffield Hospital for Sick Children was opened recently by Lady Mary Howard, Mayoress of Sheffield.—"Sister Marion" has reigned in Barton Ward at the London Homœopathic Hospital for twenty years, and a reception was held the other day at the hospital in her honour. Some handsome gifts were presented to her on this occasion from members of the hospital staff and other friends.—The new wing of the Royal Alexandra Hospital for Sick Children, Brighton, which provides new accommodation for the nursing staff, was opened on Wednesday.—On Tuesday Princess Christian devoted herself to the cause of London charities. Her Royal Highness first opened an exhibition in aid of three charities at the Jerusalem Chamber, Westminster Abbey, and afterwards visited an exhibition in Park Lane for the encouragement of Indian art.

### Hygiene: For Nurses.

By John Glaister, M.D., F.F.P.S.G., D.P.H.Camb., Professor of Forensic Medicine and Public Health, St. Mungo's College, Glasgow, &c.

### XXXII. — INFLUENCE OF CLIMATE, SEASON, LOCALITY, &c., ON INFECTIVE AND CONTAGIOUS DISEASES—MODES OF CONVECTION OF INFECTIVE MATERIAL.

Climate may be defined as the resultant of the following factors, viz.: Annual range and extremes of temperature, atmospheric pressure, humidity, prevalent winds, exposure, and certain other local conditions. In respect of diseases generally, it may be said that, in winter and spring, pulmonary ailments are most prevalent, and in summer and autumn those of the abdominal type. In respect, however, of influence of climatic on zymotic diseases, we will follow the order of the list of these already given. Small-pox prevails most in winter and spring, least in the other seasons. The temperature has a notable effect on its spread and decline. Below 50° Fahr. the disease tends to spread, above that it tends to decline. Scarlet fever prevails most in the last six months of the year, the maximum being in the December quarter. A temperature of about 60° Fahr. favours the disease, a fall to 50° Fahr. tends at once to arrest it. Measles is a disease chiefly of November, December, January, May, and June months. The most favourable temperature for its spread is between 45° and 55° Fahr., while above 60° Fahr. or below 40° Fahr. it receives a check. In 1880 Babès isolated a micro-organism—a micrococcus—which he believed to be the prime cause of the disease. Typhus fever—largely due for its inception to overcrowding and filth—is most prevalent at those seasons when the poor and uncleanly herd most closely together. It is, therefore, more common in the winter months than in summer, although there is a rise in the mortality from it in July. The infective matter, although very virulent within close range of the affected person, quickly succumbs before fresh air and sunlight. From the patient a characteristic odour is given off, which is apt to adhere to clothing. The micro-organism of typhus has not yet been isolated. There is no disease of the infective type which has receded before the march of sanitary measures more than typhus; whereas, formerly, victims could be counted by the thousand, they do not now number beyond the tens. Enteric fever is most prevalent in the autumn months. It would appear as if the climatic conditions were then most favourable for the development of the Gaffky-Eberth bacillus. It is more a disease of rural than urban communities, and epidemics in the latter are usually imported from the former. October is the month of its maximum prevalence. The bacillus of the disease is usually conveyed by infected milk or water, hence the necessity for close supervision of milk and water supplies. The infective matter is thrown off from the body of the patient chiefly in the stools, hence great care should be exercised in their disinfection. Yellow fever is confined to climates very different from that of these islands. Its range of distribution over the earth's surface is limited to the West Coast of Africa, the West Indies and Gulf of Mexico, some parts of South America, and to limited tracts of the Atlantic and Pacific sea-boards of North America. Within these limits it is usually endemic. Strangers are more liable to be seized than natives, since the latter have become acclimatised. Immunity is only established after long residence, and even then is imperfect at best. Second attacks, however, are rare. A temperature below 20°. Cent. (68° Fahr.) prevents the diffusion of the disease. It is not a contagious disease; that is to say, a person who is suffering from it, or the body of a person who has died from it, will not convey the disease to others. It is communicable, however, all the same, by infected houses, ships, and localities. It follows sea-trade routes. The micro-organism has not yet been isolated. Relapsing fever—sometimes also called bilious typhoid—is a disease which, of late years, has not been prevalent in this country. Its last visit was about twenty-five years ago. It has a remarkable coincidence, both as to time and place, with typhus, and seems, like it, to be contingent upon privation and overcrowding. Its prime cause — the *spirillun Obermeierii*—is named after Obermeyer, who discovered it. It cannot be successfully inoculated in the lower animals, except in the anthropoid apes. It is both contagious and infectious. In some epidemics laundry-women who washed the clothing of those affected were seized. Cholera is, in this and most other countries, essentially an epidemic disease, although in certain countries it is endemic. It produces a high mortality. This country has sustained four several sieges from it, viz., in 1831, 1848, 1853, and 1866, with a few isolated cases in 1894-5. The freedom of this country during the last twenty to thirty years must be mainly attributed to better water supplies and improved sanitation. It is essentially a water-borne disease. The intestinal discharges contain almost solely the infective matter. Stools, therefore, must be thoroughly disinfected, either by being mixed with sawdust and burned, or by chemical agents. The Finkler-Prior microbe and the "*comma*" bacillus of Koch have each, in turn, been blamed as the prime cause. Careful and prolonged research by various observers in different countries has established beyond reasonable doubt that the latter is the source of the mischief. Influenza has comparatively recently ravaged the countries of the world. It would appear to be uninfluenced by meteorological conditions, and climate to have as little effect as weather or seasons. It spreads with as much facility in Iceland as at the Equator, and knows no boundary by zones. It is often coincident with a disease called "pink-eye" in horses, which veterinarians believe to be influenza of horses. It would seem as if there was, at times, a causal relationship between these diseases in animals and man. It is both contagious and infectious. A microbe, said to be peculiar to the disease, has been isolated, but future research is necessary to verify or disprove this claim. Whooping-cough is commonly a disease of tender years, although by no means unknown to attack those of maturer years. Its mortality is highest among children; under one year, it amounts to about 60 per cent. of those attacked. It and measles kill more children than all the other zymotic diseases put together, and, not uncommonly, they are the sequel to one another. It is both contagious and infectious. In one isolated island in the West of Scotland, for twenty years before 1892, the disease was unknown. In that year, however, it was conveyed thither, and out of a total population of 380 persons, 114 were attacked, at all ages up to 20. An interesting fact, as bearing upon the contagious character of a common cold, has been frequently observed among the inhabitants of the remote island of St. Kilda, which is only approachable once or twice a year. Whenever a visit is paid by a ship, a large number of the islanders are seized with what they call "the strangers' cold." The micro-organism of whooping-cough has not yet been isolated. The mortality is highest in the first three and a half months of the year, and lowest in the summer months. This is doubtless due to the pulmonary complications which are more likely to arise from the inclemency of the winter months.

### Royal British Nurses' Association.

### DR. BEDFORD FENWICK'S ACTION.

Formerly, Mr. Randolph, when representing Mrs. Bedford Fenwick, used to threaten us by his letters at frequent intervals, *vide* his note of May 20th, 1889 (see The Hospital, May 25th, 1889), in which he said he was instructed to take immediate proceedings for libel whenever it may be necessary to do so; and a further one (see The Hospital for June 7th, 1890). The last one we received was dated March 1st, 1893, and appeared in The Hospital of March 11th, 1893. Probably, therefore, the following correspondence may interest our readers, especially if they will look up the Randolphian letters to which we have referred. Messrs. Mear and Fowler now represent Dr. Bedford Fenwick, and, though their letter is inordinately lengthy, we are glad to note that the language used by them is an improvement upon that formerly adopted.

"To the Editor of The Hospital.

"Sir,—We have been consulted by Dr. Bedford Fenwick concerning the publication in your paper of October 24th of what purports to be a detailed report of the General Council meeting of the Royal British Nurses' Association, held on October 16th, 1896.

"That report is, we are informed, in various particulars most inaccurate and misleading. We desire to draw your attention to that part of the report according to which Sir James Crichton Browne made the following assertion (p. 36): 'And in connection with finance, there was one statement made by Dr. Bedford Fenwick on oath which I should be glad to hear him justify. He said the Association is now £800 in debt.' We are informed that when, at the meeting in question, Sir James Crichton Browne made that statement, in somewhat different words to those reported by you, Dr. Bedford Fenwick immediately refuted it, and that Sir James Crichton Browne at once withdrew and apologised for the assertion. Yet, your report publishes that statement while it suppresses the facts of its refutation and of its withdrawal.

"Your report omits to mention the formal protest immediately entered by Dr. Bedford Fenwick against being deprived of his undoubted right to defend himself against the lengthy attack which was made upon him by Sir James Crichton Browne.

"Your report also omits the simple explanation given at once by Dr. Bedford Fenwick to Princess Christian's question respecting the nomination of the honorary officers, and thus leaves it to be inferred that Dr. Fenwick was in some way at fault in not correcting the mistake or oversight of the officials.

"Your report further omits to mention the facts that the irregular resolution re-electing Sir James Crichton Browne and the other honorary officers was put to the meeting and passed; that Dr. Bedford Fenwick entered at once a formal protest against this infringement of the charter; and that special meetings of the Executive Committee and of the General Council were hurriedly convened, and met on October 23rd to correct the irregularity.

"The misleading character of the report to which you have given publicity is therefore so evident that, on behalf of Dr. Bedford Fenwick, we must request you to publish this letter in the next issue of your paper.—We are, sir, yours faithfully,

"(Signed) MEAR AND FOWLER.

"2, Old Serjeant's Inn, Chancery Lane, W.C.,
"October 31st, 1896."

"To Messrs. Mear and Fowler.

"Dear Sirs,—Your letter of the 31st ult., addressed to the Editor of The Hospital, has been handed by him to us for reply.

"We may point out to you that the matters referred to in your letter which were not dealt with in the issue of The Hospital of October 24th, have been disposed of in its issue of October 31st, which was in the hands of the public twenty-four hours before your letter was written. In case you should not have seen The Hospital of October 31st, we send a copy by the same post as this.—Yours faithfully,

"(Signed) Baker and Nairne.

"3, Crosby Square, Bishopsgate, E.C.
"November 2nd, 1896."

The foregoing correspondence should be read in conjunction with the report of the General Council Meeting of the above Association, *vide* The Hospital for the 24th ult., and of the Special Meeting of the Executive Committee of the Association which we published on the 31st ult. It is amusing to find how ready Dr. Bedford Fenwick is to attack other people, and how he hastens to complain when the proceedings in which he takes so leading a part are fully and fairly reported.

### Mediæval Practice in the Care of the Sick.

### III.

"Phisitions . . . prescribe a remedy to each disease and bodies maledie, . . . know what is nocious and what is good; when it is fit to bath, to purge, let bloode. . . . They know the nature and the power of every simple, every herbe and flower . . . yet all their skill as follie I deride, unless they rightly know Christ crucified," says another old writer, and we have recorded many practical examples that the teaching was not vain, but that strong spirits were able to soar above their sick flesh to an amazing extent.

Ethelreade, the Saxon saint of Ely, had a tumour below her jaw; when her doctor, whose name was Cynifrid, came to lance it, she expressed lively satisfaction. "Once I used to wear vain necklaces round my neck, and now instead of gold and pearls, God in his goodness has weighed it down with this red, burning swelling."

Ethelred, a monk of the Cistercian Abbey, Rievaulx, who died in 1166, had, for a year before his death, a cough so severe that after it attacked him sometimes he was unable to speak, and "lay exhausted on his pallet."

One day when his sickness was very sore, as he sat on a mat before the fire with his head on his knees, a fellow monk came into the room, and "declaring that he was only shamming," threw him mat and all on to the fire. The other monks luckily came to the rescue, and "laid hold of the offender."

Ethelred, however, would not allow his tormentor to be punished, but kissed and forgave him.

"To cure" is the watchword of every party and of every platform in the nineteenth century; "to endure" was the maxim of the ages of faith. In the thirteenth century mankind with one accord regarded this life as the prelude to eternity; rebellion against corruption, against abuse of the temporal power, there was in plenty, but in spiritual matters the Church was recognised as the ultimate authority against whose decisions there could be no appeal.

Three centuries of independence, free thought, or whatever individual bias may dispose us to call the movements which originated in the sixteenth century, have made all theological subjects—eternity amongst them—such vexed questions that, in our day, the magic word "philanthropy" is hailed with delight as the expression of, at least, one interest common to all Englishmen alike.

In the name of love for humanity, a Cardinal of the Holy Roman Church will sit side by side with a Freemason, the Archbishop of Canterbury will shake hands with a Baptist minister, and a Scotch Presbyterian will agree with an Agnostic in the board-room of a hospital upon questions relating to the relief of their disease-stricken neighbour.

The ideals of the Middle Ages were different. Early English theologians believed firmly that fleshly ills might conduce to the soul's health, and hence it is not surprising to find few traces in their writings of that feverish desire which animates us to be rid of anything and everything, disease included, which is disagreeable. The influence of a steadfast belief in a life beyond the grave is strongly marked both in the conduct of the sick people themselves and in that of their nurses and doctors. Hospitals founded now are intended primarily to cure diseases of the body; those of the Middle Ages were rather places of refuge for persons needing preparation for the long journey whence no traveller returns. When "to visit the sick" was enjoined by directors upon their penitents, it was the care rather than the cure of the sufferers which was intended.

### Nurses in 1896—Their Quarters, Hours, and Food.

[These articles exhibit the actual condition of affairs in the spring of the present year.]

### KING'S COLLEGE HOSPITAL.

### I.—Terms of Training.

Probationer-nurses and special probationers are received for training at King's College Hospital, the qualifications, duties, and training of the latter being in all respects the same as those of a probationer-nurse. Applicants for appointment must be not less than twenty-five nor more than thirty years of age. They are engaged for a term of three years, receiving a certificate at the end of that time. Probationer-nurses are not paid during their first year. At the termination of the three years' engagement a nurse may, if approved by the committee of management, renew her engagement on terms of one month's notice on either side, binding her to continue as nurse at the disposal of the committee in the hospital.

Special probationers pay for their training ten guineas a quarter for the first year and five guineas a quarter for the second. At the end of the second year, if approved by the committee, a special probationer is appointed unpaid staff nurse.

From November to June each year lectures on anatomy, physiology, medical, obstetric, and surgical nursing are given by members of the medical staff on Fridays, and classes are held by the Home Sister in connection with this instruction. Examinations are held during the first and second year's training. The certificates given are of varying grades, so as to embrace those practical nurses who show to greater advantage in the wards than on examination papers, for at King's it is not believed that the nurse who obtains the highest number of marks is necessarily entitled to the greatest commendation as a good all-round nurse, and for this reason medals and prizes find no place in the nursing curriculum at King's College Hospital.

### II.—Hours of Work and Times off Duty.

The work at King's College Hospital is arranged after a carefully thought out and mathematically planned system of Miss Monk's own invention, and the actual hours of the nurses on duty are less than at any other London Hospital. The day staff come on duty at seven a.m. and go off at nine p.m., during which time every nurse is away from the wards for four consecutive hours, besides other meal times, bringing the working hours, approximately speaking, down to nine. The night nurses are on duty from nine p.m. to nine a.m., having two half hours off during the night for meals at definite times. The nursing staff at King's in proportion to occupied beds is large, about one nurse to two and a half beds. In a ward of thirty beds, for instance, on day duty the staff consists of sister, one staff nurse, and four probationers; on night duty one staff nurse and one probationer. Every nurse is off duty for four consecutive hours each day, and it is considered to be as great a crime to be a minute late in leaving the ward as in returning to it. The remarkable absence of illness amongst the nurses is doubtless due to the ample opportunities for rest and exercise given to them. The off-duty times are taken in a regular order or rotation, which can only be changed by Miss Monk's direct permission, and are arranged on a plan which, by her permission, we give in detail :—

Plan of Nurses' Four Hours-Off Duty.

| Hours off Duty. | Monday. | Tuesday. | Wednesday. | Thursday. | Friday. | Saturday. |
|---|---|---|---|---|---|---|
| 7 a.m. to 11 a.m.... | Pro. 1 | Pro. 2 | Pro. 3 | Pro. 4 | Pro. 1 | Pro. 2 |
| 10 a.m. to 2 p.m.... Do. | Pro. 2 | Pro. 3 Staff Nurse. | Pro. 4 | Pro. 1 Staff Nurse. | Pro. 2 | Pro. 3 Staff Nurse. |
| 2 p.m. to 6 p.m.... | Pro. 3 | Pro. 4 | Pro. 1 | Pro. 2 | Pro. 3 | Pro. 4 |
| 5 p.m. to 9 p.m.... | Pro. 4 | Pro. 1 | Pro. 2 | Pro. 3 | Pro. 4 | Pro. 1 |
| 6 p.m. to 10 p.m.... | Staff Nurse. | | Staff Nurse. | | Staff Nurse. | |

Sundays are worked on the same plan.

Probationers are numbered 1, 2, 3 and 4, and change their numbers every Monday morning. The diagram shows order of rotation.

It will be seen that the scheme is worked backwards, by which an advantage is gained by probationers 1 and 2, that is, a long night's rest twice a week, and as each nurse becomes in turn No. 1 and 2, all probationers benefit.

The object of the early hours off duty in the morning is with a view to allow an occasional long night's rest, probationers in their first two years who usually suffer much from unaccustomed standing, being especially provided for in this way, remaining in bed if they choose from half-past ten p.m. to half-past eight the next morning, when they are called, and come down to a comfortable hot breakfast at half-past nine, going to their wards at eleven a.m. It is thought to be good for all concerned that nurses should be given every reasonable facility for mixing with the outside world, with the belief that they come back to their work all the brighter and better for so doing. The hours are, therefore, so arranged that when off duty nurses may be able to go out to lunch, tea, or dinner with their friends. Probationers are allowed one whole day off duty each month, from half-past six a.m. to ten p.m.; staff nurses on day duty have monthly leave of absence from Saturday, half-past four, to Monday, ten a.m.; night nurses have two nights every month; and the sisters' monthly leave is from Saturday, half-past four, to Monday, two p.m.

The yearly holidays are three weeks during the first and second years, for the third and subsequent years four weeks, while the sisters have six weeks' holiday in the year.

### III.—Meals.

The food provided for the nurses is thoroughly good, and there is no limit in amount in the way of "allowances." The dietary is much the same as at other hospitals. Tea and coffee, bread and butter, eggs and bacon, potted meats, and so forth being the rule for breakfast; fresh meat, two vegetables, and pudding for the chief meals of night and day staff, with a certain allowance of beer, ale or stout, or milk. Tea is served in the dining-room in the afternoon for the nurses; the sisters have their breakfast and tea in their own rooms. Sister Katharine (as Miss Monk is best known to her nurses) dines in the middle of the day at one of the two dinners with the sisters and nurses, and has supper with the sisters alone in the evening. For the ward meals at night special arrangements are made for each nurse to be off duty for half an hour twice in the night. At twelve o'clock a light meal of tea or coffee and bread and butter is taken in the ward kitchen, and at four o'clock a second meal of a more substantial character, with cold meat, bacon, or haddock. The provisions for these meals are taken each night from the dining-room to the ward kitchen. No meals are permitted in the wards under any circumstances, and the nurses are expected to sit down to a properly-laid table, and to take half an hour, the probationer first preparing the nurse's meal and afterwards taking her own. Special care is taken at King's that the table linen shall be fresh, and the meals well served in every case, and that the tables shall be made cheerful with flowers and plants. It is on this point that so often sufficient stress is not laid, for food, however good in itself, if served to the accompaniment of crumpled tablecloths and dull glass must always be unappetising.

### IV.—Salaries and Uniforms.

Probationer-nurses are not paid during their first year. For the second year they receive a salary of £15. At the end of the second year they may be appointed staff nurse, and for the third year are paid £20, payment thereafter rising in the fourth year to £30, fifth year £33, and sixth year £36. Sisters' salaries range between £35 and £50. Uniform is supplied by the hospital to all the staff, including special probationers. Three print dresses are allowed yearly, and one stuff (grey alpaca) for outdoor wear, with caps and aprons as required. The sisters wear "Raphael green" merino dresses, of which they are provided with two in the year. Outdoor uniform is also provided by the hospital, and nurses are expected always to wear this, except when special permission is given for "worldly" garb to be substituted. All nurses have an allowance of 2s. weekly for washing.

### V.—Nurses' Quarters.

King's College Hospital possesses no separate nurses' home; the quarters for the nursing staff are on the top floor of the hospital itself. Here are cubicles—excellent of their kind, and very comfortable—for nurses and probationers, and two sitting-rooms for their use. The sisters are accommodated with pleasant bed-sitting-rooms. More space is much needed for additional sitting-rooms, and Sister Katharine is endeavouring personally to raise a sufficient sum of money to carry out certain improvements in this way for the accommodation of her staff, over whose comfort she spends much thought. The dining-hall, a good-sized apartment, is on the basement floor, but is a well-lighted, airy room, looking cosy enough at meal-times, with its nicely-appointed tables. There is only one dining-room, the sisters sitting together at one table, and Miss Monk herself taking her meals with her nurses. The matron's sitting-room and office, and the home sister's office and linen-room are on the first floor of the hospital.

### Hints to Nurses Going to the Riviera.

By One Who Has Been There.

At this season of the year, when delicate persons are thinking of moving off South in search of sunshine, a few hints as to the principal invalid resorts may be of service to nurses who have charge of such patients. First and foremost, among such winter quarters, ranks the Riviera. But the Riviera is not a place, it is a district, a long line of seaboard stretching along the Mediterranean Sea, each station of which has its own distinguishing characteristic. And these characteristics are due to local conditions, such as the position in relation to the mountains behind them, &c. This, therefore, is a question to be settled between doctor and patient, which is the particular place best suited to the requirements of the case. Hyères, Mentone, Bordighera, and other smaller stations on the Italian Riviera, are the principal health resorts, whilst Monte Carlo, Nice, Cannes, and San Remo are perhaps popular on more social grounds, though the two latter named combine the characteristics of each. The seasons are from November till April, but delicate persons are wise not to return to England until the first of the May winds are over. The good gained by a winter of sunshine abroad has often lost its value by a too hasty return to the English climate, which is not generally in a very settled condition till May is well on. We have just spoken of a winter of sunshine on the Riviera, because really anyone who knows anything about it practically knows that it is sunshine rather than actual warmth which recommends the place. Think a moment what it means when we consider that whilst London has an average of twelve cloudless days during the six winter and spring months, Nice has 97! Yet, taking it all round, the average mean temperature during winter is not more than 10 degrees higher than in England. What really distinguishes the Riviera, is its abundance of sunshine, its mild temperature, exemption from rainy days, and its stimulating atmosphere. Of course it rains there; but, after all, we must bear in mind the difference which exists between rainy days and the actual rainfall. A word or two must be said as to the cost of living, and as to the expenses of the journey. Persons with very moderate means can spend a winter by the Mediterranean without incurring any serious expense. I say they can, and, indeed, they do do so, though there are no limits to possibilities for the outlay of money, to those who are well endowed with this world's goods. But the Riviera is for the poor man as for the rich, and one can be very comfortable on any of the stations on quite ordinary means. The Italian Riviera is really the cheapest to stay at, and it is much less expensive than an English wintering resort. A very reliable authority on this question declares that the further the traveller goes East after leaving Mentone the more moderate are the charges. The usual route to the Riviera is viâ Dover, Paris, and Marseilles. For the convenience of easy reference to those about moving southwards, Messrs. Cook and Sons have prepared a book of valuable information concerning the ordinary and express routes, daily services, train fares, &c., which can be obtained at any of their central depôts, and to which we beg to refer our readers.

### Everybody's Opinion.

### NURSES' CO-OPERATIONS.

Nurse Mary Hartley writes: Seeing in The Hospital that a correspondent is inquiring whether you know of any other nurses' co-operations other than that in New Cavendish Street, permit me to draw your attention to the North London Co-operation. At present we are only twelve, but, of course, as we get more work our staff will increase. Our superintendent opened this last October, and since then we have done better than we even expected. I joined in January, and have since earned £53, and have only been three weeks without a case. The rules and regulations are as near as possible to those of the New Cavendish Street Co-operation, which we consider the only genuine one. We have only co-operation nurses on the staff—no salaried ones. We pay our superintendent 3s. 6d. per week all the year round instead of the usual percentage. There is no other expense except our board when in the home (10s. 6d. per week). Our superintendent takes only nurses who have obtained full three years' training, and can give her a matron's reference. The doctors round the neighbourhood, also at a distance, have been pleased to give us their work, also several good and successful operations in the home.

[An institution "run" by a private individual for profit cannot call itself a "co-operation." The papers enclosed in the above letter do not show that the "North London Co-operation" has any committee, or that it is other than a private venture.—Ed. *T. H.*]

### ORPHANS CAGED.

A Medical Correspondent writes: It is difficult to take seriously one who, like the medical officer of St. Mary's Home, Broadstairs, does not address himself to contested points, but indulges in correspondence "full of sound and fury signifying nothing." The whole tone of this gentleman's animadversions reminds me of the forensic story of the solicitor for the defendant who instructed his counsel, "No case; abuse the plaintiff's attorney." But it may be well to point out to him that abuse is not argument, and that scurrility is a sorry substitute for sound sense. As he appears to be of a poetical turn of mind in quoting Lovelace's opinion that "iron bars do not make a cage," may I commend to him a few lines from Hamlet? who says, truly enough:

> "Rightly to be great
> Is not to stir without great argument;
> But greatly to find quarrel in a straw,
> When honour's at the stake."

Mr. Raven has thought fit to impugn my honour, and in order to sustain his point has imputed to me insinuations which are certainly not involved in my statements, and can only emanate from his own ingenuous (?) imagination. Where, I would ask him, did I suggest that the cubicles commented on contained "straw scattered over bare boards," or that there was anything "wet and dirty" about them? What I did object to, and what I still object to, as unworthy of a nineteenth-century Christian sisterhood, is the fact that poor infants committed to their care are at night "cribbed, cabined, and confined each within its own enclosure of iron bars, from which no escape is possible till the clips which fasten the prison doors are withdrawn." As regards the resemblance of the so-called cubicles to "the cages of a travelling menagerie," I have appealed to the judgment of your readers, who have been aided to form an opinion by the reproduction of a sketch (made at the parent institution) of similar "cubicles," which, if "rough," has not been alleged to be inaccurate, and fairly represents my impression of what I saw, and what I said I saw, at St. Mary's, Broadstairs. The word "abomination" nowhere occurs in my correspondence; that is an epithet for which Mr. Raven himself is responsible, however appropriate it may be to the state of the case. In conclusion, I would remind Mr. Raven that distorting statements and imputing "wilful misrepresentation" is hardly such treatment as one would expect on the part of a "practitioner of thirty years' standing," even towards a medical correspondent who prefers to remain anonymous, though possibly of equal professional status with the Medical Officer of St. Mary's Home.

### COUNTY COUNCIL GRANTS AND NURSING.

A Correspondent writes: I notice that the question of how far and in what manner it is legal for County Councils to make grants in aid of "instruction in nursing" has been agitating the County Council of East Suffolk. It would seem that "nursing scholarships" of £20 or £30, providing training for six months at some hospital, are allowable, but not direct grants to district nursing institutions. Now it seems to me that the suggestion of Colonel Moore, recently made in a letter to the *County Council Times*, would, if adopted, lead to far more useful results than this encouragement on the part of the County Council of half-trained women who will flood the country calling themselves nurses. Colonel Moore says, "There is one subject which must benefit all classes and all ages of the community—better nursing. . . . Can a portion of the money be more advantageously spent than by giving practical knowledge in the simpler principles of nursing? Nursing may be supplemented by lectures, but not actually taught without ocular demonstration. Such demonstration is impracticable in a rural district without a hospital, and the only way of imparting such knowledge is by having a qualified nurse located in it who can attend the sick in their own homes and impart her knowledge to the family and neighbours who live with or near the sufferer." District nurses are the best instructors of the poor, for they teach them the first principles of cleanliness and health; but it is eminently necessary that these instructing nurses should be well-educated, thoroughly-trained women themselves. Interpreting thus the word "instruction" to mean the teaching of the people in their own homes, not the provision of a short and insufficient training in nursing of women chosen from the country districts, might not the payment of a district nurse, carefully selected for her capability to teach as well as nurse among the poor, be recognised as legitimate expenditure on the part of County Councils? I should like to hear what those who have had experience in the matter think would best tend to the instruction of the people generally—the provision of one thoroughly-trained "nurse instructor," to act as a health missioner as well as nurse, or the giving three women each six months' experience in a hospital and turning them into country villages to nurse the sick with this very limited knowledge?

### Nursing in the Colonies.

### COLONIAL HOSPITAL, SUVA, FIJI.

The editor has been asked to select an associate nurse for this hospital. The qualifications are: "A good, practical, hardworking nurse, with a sound professional training, a robust physical constitution, and an even temper, competent to perform the midwife's functions in cases of natural labour, and other non-instrumental cases. L.O.S. certificate and previous tropical experience, especially in India, desirable." The editor will be glad to receive applications from any nurse with Indian experience, possessing the L.O.S. certificate, accompanied by copies of not more than three testimonials and a short statement as to previous experience and training. Applications should be marked "Fiji" in the left corner of the envelope.

### Minor Appointments.

North Lonsdale Hospital, Barrow-in-Furness.—Nurse M. L. H. Warner has been appointed Charge Nurse at this hospital. Nurse Warner was trained at the Sussex County Hospital, Brighton.

Carlisle Workhouse Hospital.—Miss Edith Amy Hempstock has been appointed Head Nurse at this hospital. She was trained at St. Mary's Hospital, Manchester, where she afterwards held the position of staff nurse. Miss Hempstock left St. Mary's to join the Oldham Nursing Association as nurse, and was then appointed head nurse at Greenwich Union Infirmary.

### Wants and Workers.

Will anyone lend a copy of Burdett's "Hospital Annual" for 1894 to "Nurse, Ravendale, Weybridge"? Postage will be returned.

### Where to Go.

Cheyne Hospital for Sick Children, Chelsea.—A sale of needlework will be held at the Cheyne Hospital, Cheyne Walk, on Wednesday and Thursday, November 25th and 26th, in aid of the Lawrence Street Annexe Fund. Admission on Wednesday, two to six o'clock, 2s.; on Thursday, two to eight o'clock, 1s.; six 2s. tickets 10s. 6d., twelve for £1; twelve 1s. tickets for 10s. Tickets may be obtained from the hospital.

Froebel Educational Institute, Palgarth Road, West Kensington.—A course of twelve lectures on "Points in the Special Training of Backward and Mentally-Feeble Children" is being given at the Institute by Dr. G. E. Shuttleworth (formerly Medical Superintendent of the Royal Albert Asylum, Lancaster.) The lectures are on Thursday afternoons at half-past four, the last of the first half of the course being on December 10th. The second half begins on Thursday, January 21st, and continues on Thursday afternoons to the end of February. Applications for admission to the lectures to be made to Madame Michaelis, at the Institute, from whom all information can be obtained. Fee for the course £1 1s., for each of the half courses, separately, 15s.

### Appointments.

### MATRONS.

St. Olave's Union Infirmary, Rotherhithe.—Miss Augusta M. Orchard has been appointed to fill the position of Matron at this infirmary. Miss Orchard was trained at the Chorlton Union Hospitals, Withington, Manchester, where she was subsequently promoted to be charge-nurse. For the last five and a-half years she has held the appointment of Superintendent of Nurses at the Fusehill Hospital, Carlisle.

Dewsbury and District General Infirmary.—Miss Elicia Nunn has been appointed to the post of Matron at this hospital. She trained at the Halifax Infirmary, where she was afterwards promoted to be staff-nurse. Miss Nunn then joined the staff of the Dewsbury Infirmary as charge-nurse, from which position she has been promoted to that of Matron.

Wantage Cottage Hospital.—Miss Sarah Evans, of the Frome Cottage Hospital, has been appointed Matron of this hospital in succession to Mrs. Curnow, who has retired on account of advancing age. Miss Evans was trained at the Wolverhampton General Hospital and the Royal Albert Edward Infirmary, Wigan. She was charge-nurse at the latter institution for five and a half years, senior charge-nurse at the Dudley Guest Hospital for two years, and has been matron of the Frome Cottage Hospital for four years.

### Novelties for Nurses.

Messrs. D. H. Evans and Co. (Limited), Oxford Street, have recently added a fresh department to their ever-increasing premises for the convenience of nurses. A most useful stock of linen aprons in all sizes and shapes are now being offered at such low prices that one pauses to consider whether they can be remunerative. The secret, however, has crept out, and it is so highly creditable to the firm that we trust they may be encouraged in their enterprise by the demand we confidently anticipate will be their reward. At their Irish factory during the busy season a large number of hands are employed, and to avoid turning them off when ordinary business becomes slack it has been decided to keep them on and direct their energies from the manufacture of underlinen to that of cap and apron making, giving the customer the benefit of the profit that arises from direct supply. These aprons are not only well cut and beautifully made, but are of ample width, an advantage not often met with in the cheaper sorts of ready-made articles. Cuffs and collars of the popular "Squire" shape can be had either plain or hemstitched, the latter presenting a specially dainty appearance. There are also a large variety of caps, all of which are drawn into shape by a string, which unties for washing. Before long the department will include dresses, cloaks, and bonnets, which will largely add to its prospect of future success.

### Notes and Queries.

*A Domestic Problem.*

(43) Do you think it is ever desirable that the doctor of a small hospital, sometimes without patients, should live *en famille* with the sisters? This plan has been adopted at my hospital, and I should be glad to hear views for and against it.—*Matron.*

Perhaps some of our readers will give our correspondent the benefit of their experience on this subject.

*Appointment Wanted.*

(44) I should like to meet with an appointment as matron in a small hospital in New Zealand. I am fully qualified. Would you advise me to go out and try?—*S. J. H.*

Many nurses would "like" to find a small hospital just suited to their requirements, but those who are unwise enough to migrate to a strange land "to try" for such desirable posts generally find themselves in very undesirable positions. Colonial hospitals train plenty of good nurses, and English nurses are by no means in such demand as they appear to imagine. We are for ever warning nurses against leaving England without a certainty of employment.

*Children's Nurses.*

(45) Can you tell me if there is any recognised institution for training ladies as children's nurses?—*Medicus.*

There is the Norland Institute, 29, Holland Park Road, where ladies are given nine months' training (in which is included three months' hospital training), to fit them for the charge of children. There is now a considerable demand for Norland Institute nurses.

*Monthly Nursing.*

(46) "A Midwife" would be glad to know if there is any institution in the United Kingdom which would employ her as a monthly nurse without general training. She has passed the L.O.S.

Why does not our correspondent advertise? She might write for advice to the Secretary of the Midwives' Institute, 12, Buckingham Street, remembering to enclose a stamped envelope for reply.

*Dispensing.*

(47) Is there an opening for women dispensers, and what is the best training?—*Nurse.*

You will find the subject fully dealt with in Burdett's "Hospital Annual for 1894." Miss Bradbury, resident dispenser at the Ryde Dispensary, Isle of Wight, takes resident pupils into her house, the usual course of instruction extending over six months, or longer, at the pupil's option. You might write to her. There is certainly an opening for women as dispensers.

*Latin for Nurses.*

(48) Please tell me what Latin book a nurse may study with advantage, and if it is necessary to go through the whole Latin grammar?

Very certainly a nurse does not "require" to go through the whole Latin grammar! A knowledge of Latin is useful to anybody, but to know how to make beds, sweep a room, and prepare an appetising meal is of far greater importance to the would-be nurse than an acquaintance with a dead language. How to read and write her own intelligently is as much as will be expected from her in the matter of languages.

*Quarantine.*

(49) Is there any home in London where private nurses are received after nursing infectious cases?—*Nurse.*

We believe Miss C. J. Wood, Nurses' Hostel, Percy Street, Tottenham Court Road, takes nurses in this way.

*Anatomical Illustrations.*

(50) I shall be much obliged if you can give me the names and addresses of some firms who supply drawings of the various organs of the human body with explanatory letterpress.—*C. Board.*

You had better write to Messrs. Baillière, Tindall, and Cox, King William Street, W.C. They publish a work entitled, "Human Anatomy and Physiology," illustrated by a series of movable atlases of the human body, showing relative positions of the several parts by means of coloured plates. They also have White's "Physiological Mannikin," showing all the organs of the body, life size, and they have, besides these, other less expensive books on anatomy with illustrations.

*Poor Law Officers' Superannuation Act.*

(39) Will you tell me if in "contracting out" of this Act, it holds good if a nurse takes another appointment, or would she under another board of guardians fall under its provisions? I am the only nurse in a Union Infirmary, and have no one to confer with me on this matter, and shall be therefore very glad of your advice.—*Subscriber.*

In section 15 of the Superannuation Act, concerning "Existing Officers and Servants," it is stated that any officer or servant in the employment of guardians or other authority to whom the Act applies can "signify in writing to such authority his intention not to avail himself" (or herself) "of the provisions of this Act, and in that event it shall not be obligatory on him . . . . to make any contribution or submit to any deduction from his salary or wages under this Act . . . . ," and that such officer or servant "shall remain subject to the provisions of the Poor Law Officers' Superannuation Act of 1864,"&c. If you avail yourself of this clause we believe that you will continue subject to the conditions under which you originally took service in the Poor Law for so long as you shall remain in it. If you neglect the opportunity of contracting out before the end of this year you will be compelled to contribute to a fund of which, as a nurse, you are never likely to benefit, inasmuch as nurses seldom or never remain at work until they are sixty years of age. There is no question but that in her own interests every nurse at present in the service of the Poor Law should protect herself in this way from an Act which is most unjust to nurses and indeed to all woman who work under the Poor Law.

## Figures and Tables

**Figure f1:**